# The LysoPS/GPR174 axis drives metastatic progression in esophageal squamous cell carcinoma through cAMP-PKA-CREB signaling activation

**DOI:** 10.1186/s12967-025-06419-0

**Published:** 2025-04-14

**Authors:** Rong Xiao, Pei Xu, Xiangyuan Li, Feng Shen, Shuangfen Tao, Xiaocen Zhu, Yu Cai, Zhuowei Feng, Zhiyi Liu, Haibo Xiao, Fangbao Ding, Meiling Zhu

**Affiliations:** 1 Department of Oncology, Xinhua Hospital Affiliated to Shanghai Jiao Tong University School of Medicine, Shanghai, 200092 China; 2 Department of Cardiothoracic Surgery, Xinhua Hospital Affiliated to Shanghai Jiao Tong University School of Medicine, Shanghai, 200092 China; 3 Department of Gastroenterology, Xinhua Hospital Affiliated to Shanghai Jiao Tong University School of Medicine, Shanghai, 200092 China; 4 Core Facility of Basic Medical Sciences, Shanghai Jiao Tong University School of Medicine, Shanghai, 200025 China

**Keywords:** GPR174 protein, Lysophosphatidylserine, Esophageal squamous cell carcinoma, Neoplasm metastasis, Epithelial–mesenchymal transition

## Abstract

**Background:**

Esophageal squamous cell carcinoma (ESCC) is a highly lethal malignancy with a 5—year survival rate of less than 20%, largely due to its high propensity for metastasis and recurrence. There is an urgent need to identify targeted therapeutic agents for this disease. While lysophosphatidylserine (LysoPS) and its receptor GPR174 are known regulators of immune and inflammatory processes, their mechanistic role in ESCC progression remains unexplored. This study investigates the LysoPS/GPR174 axis in driving ESCC metastasis and its underlying molecular pathways.

**Methods:**

LC–MS was used to measure LysoPS concentration, and Western blotting was performed for protein quantification. The correlation between GPR174 expression and ESCC prognosis was analyzed using ESCC tissue microarrays, immunohistochemistry, and Kaplan—Meier survival analysis. Wound healing and Transwell assays were carried out to evaluate the migratory and invasive capacities of cells. The proliferative ability of ESCC cell lines was assessed with the CCK-8 assay. Nuclear—cytoplasmic extraction assay was conducted to separate the nucleus and cytoplasm. Metastasis model of nude mouse was employed to investigate the metastasis of ESCC cell lines.

**Results:**

We found that the levels of LysoPS were significantly increased in metastatic ESCC tissues compared to nonmetastatic ESCC tissues. Moreover, a correlation was established between LysoPS-mediated tumor metastasis and GPR174 expression in ESCC. Our results also revealed that high expression of GPR174 in ESCC is associated with tumor metastasis and poor survival outcomes in ESCC patients. Further exploration of the underlying mechanism showed that LysoPS stimulates the up- regulation of GPR174 expression. The increased GPR174 then activates the cAMP-PKA signaling pathway. Subsequently, the active subunit of PKA translocates into the nucleus, where it phosphorylates CREB, thereby promoting the metastasis of ESCC. In vivo, GPR174 overexpression increased metastasis burden.

**Conclusions:**

Our study demonstrates that the LysoPS/GPR174 axis, through the cAMP-PKA-CREB pathway, plays a crucial role in promoting the invasion and metastasis of ESCC. This highlights its potential as a novel target for predicting ESCC progression and may offer new insights for the development of targeted therapies for this deadly disease.

**Graphical Abstract:**

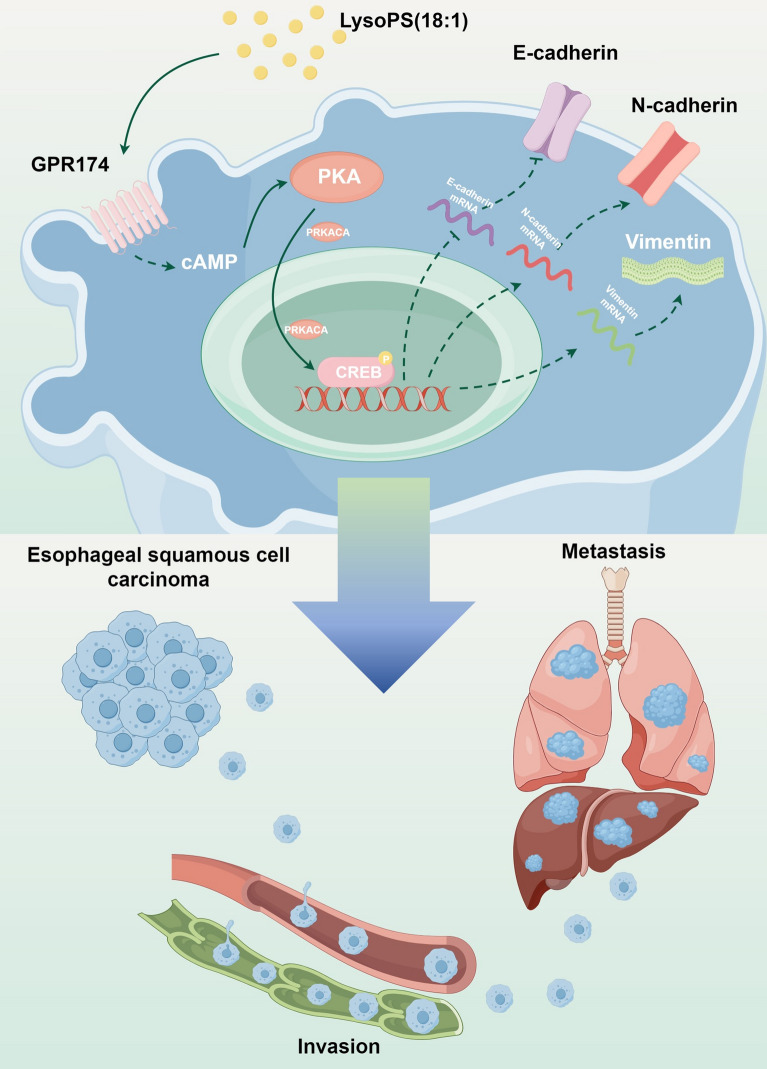

**Supplementary Information:**

The online version contains supplementary material available at 10.1186/s12967-025-06419-0.

## Introduction

Esophageal squamous cell carcinoma accounts for over 90% of esophageal cancer cases; ESCC is prevalent in South America, Asia and East Africa [1]. LysoPS, a type of lysophospholipid, is produced by the action of phosphatidylserine phospholipase A (PLA) during cellular activation or death. It has emerged as a recognized agonist and ligand for G protein-coupled receptor 174(GPR174) [2, 3]. In the P2Y receptor family, GPR174, also called LPS3, is an X-linked G protein-coupled receptor. It is abundantly expressed in the human immune system and is highly abundant in B lymphocytes and T lymphocytes [4, 5]. GPR174 is a receptor that relies heavily on the G protein alpha subunit (Gαs) for its functions, such as the negative regulation of naïve T-cell activation [6, 7].

Previous studies have shown that LysoPS and GPR174 predominantly play significant regulatory roles in inflammatory and immune-related diseases. However, recent findings indicate that LysoPS also contributes to the pathogenesis of gastric and colorectal cancers [2, 8–12]. Nevertheless, no studies have assessed the role of GPR174 in cancer, and we still do not fully understand how LysoPS/GPR174 contributes to ESCC. Cyclic adenosine monophosphate (cAMP), a second messenger, is responsible for a range of biological pathways, including those linked to malignancy [13]. Research has demonstrated that LysoPS is capable of increasing intracellular cAMP levels through the activation of Gαs proteins [14]. cAMP can activate a variety of effectors, among which one of the most studied is protein kinase A (PKA) [15]. PKA, a protein predominantly localized in the cytoplasm, is a key intracellular signaling molecule. PKA is a tetramer formed via the dimerization of 2 regulatory subunits. Each regulatory subunit contains a catalytic subunit. The catalytic subunit is inactive until it is released [16]. The regulatory subunit of PKA inhibits the activity of PRKACA, the catalytic subunit of PKA. After receiving an upstream signal, cAMP activates PKA and liberates the catalytic subunit, PRKACA, from its regulatory subunit. This freed catalytic subunit further activates downstream signaling pathways [17]. Nuclei contain cyclic AMP response element-binding protein (CREB), a transcription factor. CREB, an important downstream factor of PKA, can be phosphorylated by PKA [18, 19]. CREB is thought to promote tumor growth and metastasis and is often overexpressed and hyperactive in cancer patients [20, 21]. Recent studies suggest that CREB activation is associated with enhanced invasiveness and metastatic potential in ESCC [22]. To date, it is unclear whether the activation of cAMP-PKA-CREB plays an essential role in ESCC progression.

In this study, we demonstrated for the first time that LysoPS/GPR174 facilitates the invasion and metastasis of ESCC. Mechanistically, after activation of GPR174 by LysoPS in ESCC, the promotion of tumour metastasis by GPR174 is mediated through the cAMP-PKA-CREB signaling pathway. In conclusion, we present novel insights into the clinical diagnosis of ESCC and potential new treatment options.

## Methods

### Cell culture

The ESCC cell lines (EC-109, EC-9706, TE-12, TE-1, and KYSE-150) were graciously provided by the research group of Dr. Yanyun Zhang at the Shanghai Institute of Nutrition and Health, Chinese Academy of Sciences. Penicillin/streptomycin and fetal bovine serum (FBS) were added to RPMI-1640 medium to grow ESCC cells (medium: FBS: penicillin/streptomycin = 100:10:1). At 37 °C with 5% CO_2_, the cells were maintained in an incubator.

### Tissue samples and patients

Tissue microarrays (TMAs) of ESCC patient samples were purchased from Shanghai Outdo Biotech Company (Shanghai, China). Samples obtained from 98 patients with full clinicopathological data were utilized for statistical analysis.

Fresh ESCC tissue samples were taken from patients who had not received any preoperative treatment prior to surgery or endoscopic resection at Xinhua Hospital Affiliated to Shanghai Jiao Tong University School of Medicine, between September 2023 and February 2025. All the patients signed informed consent. The case data used in this study were fully desensitized at the time of extraction to strictly protect patients' private information. A − 80 °C freezer was used to store these tissue samples. Approval for the study was obtained from the Ethics Committee of Xinhua Hospital Affiliated to Shanghai Jiao Tong University School of Medicine.

### H-89 formulation

*N*-[2-(p-bromocinnamylamino)ethyl]-5-isoquinolinesulfonamide·2HCl hydrate (H-89)(#S1643, Beyotime) was dissolved in dimethyl sulfoxide (DMSO), and the cells were then stimulated with 10 μM H-89 for 48 h. The other two groups, the Vector and GPR174 OE groups, were exposed to DMSO without H-89 for 48 h.

### Immunohistochemistry (IHC)

The TMA was stained following the standard IHC protocol. The primary antibody used for IHC was anti-GPR174 (1:250, ab92796, Abcam). IHC scores for the TMA were assessed independently by two researchers. The percentage of positively stained cells was divided into five categories: < 5% was 0, 6–25% was 1, 26–50% was 2, 51–75% was 3, and > 75% was 4. The intensity of immunohistochemical staining was divided into five categories according to conventional criteria: negative was 0; weak was 1; moderate was 2; or strong was 3. Five random fields of view were selected, and the average count was calculated. Next, the percentage of positively stained cells and the intensity of immunohistochemical staining were multiplied to obtain a final IHC score (Fig. S1).

### Western blotting

Cell samples were lysed with RIPA buffer (Beyotime, Shanghai, China) containing protease inhibitor (Beyotime, Shanghai, China) for 25 min on ice. After adding Loading Buffer, the lysates were boiled at 95 °C for 10 min. Protein concentrations were quantified using the Bradford method, followed by electrophoresis on SDS-PAGE gels for 90 min and transfer to a nitrocellulose (NC) membrane (Cytiva, Marlborough, USA) at for 70 min. The membrane was blocked with 5% non-fat milk in TBST for 1 h at room temperature. The following primary antibodies were used for conventional Western blotting: anti-GAPDH (#60004-1-Ig, Proteintech), anti-GPR174 (#ab92796, Abcam), anti-MMP-9 (#10375-2-AP, Proteintech), anti-Lamin A/C (#10298-1-AP, Proteintech), anti-p-CREB (#9198, CST), anti-N-cadherin (#22,018–1-AP, Proteintech), anti-Vimentin (#22031–1-AP, Proteintech), anti-PRKACA (#4782, CST), anti-E-cadherin (#20874-1-AP, Proteintech), anti-α-Tubulin (#66031-1-Ig, Proteintech), anti-CREB (#9197, CST), anti-MMP-2 (#10373-2-AP, Proteintech). HRP-conjugated anti-IgG antibodies linked to (1:3000, Beyotime, Shanghai, China) were used as secondary antibodies. Protein bands were detected by incubating with ECL substrate (Millipore, Billerica, USA) and visualized using a chemiluminescence imaging system (Cytiva, Marlborough, USA).

### Liquid chromatography–mass spectrometry (LC‒MS)

Measurement of LysoPS concentrations was performed with reference to previous studies [23]. The LysoPSs used in our study were LysoPS (18:1) (#858143P, Avanti Polar Lipids) and LysoPS (18:0) (#858144P, Avanti Polar Lipids).

### Infection of Lentivirus

Lentiviruses of GPR174 were purchased from Genechem (Shanghai, China). GPR174 expression was upregulated via overexpression lentivirus, and a stable cell line with reduced GPR174 expression was established via the use of GPR174 shRNA. All the infection procedures were carried out according to the guideline of manufacturer. It should be noted that in animal experiments, we further interfered the lentivirus of luciferase into the cell. Table S1-2 lists the sequences of the lentiviruses used for overexpression and knockdown.

### Assays for cell proliferation

ESCC cell lines were inoculated in 96-well plates at a density of 5 × 10^3^ cells/well, and 10 μL of CCK-8 buffer (#CK04, Kyushu Island, Japan) was added to each well on days 1, 2, 3, and 4, respectively, followed by incubation in a cell culture incubator for 1.5 h. Then the absorbance at 450 nm was measured using a microplate reader (BioTek, Vermont, USA).

### Migration and invasion assay

ESCC cells in the upper chamber (10 × 10^4^ cells/well for the invasion assay and 5 × 10^4^ cells/well for the migration assay) were suspended in 120 μL of FBS-free medium, and the lower chamber was filled with 600 μL of medium (containing 20% FBS). 2 days later, ESCC cells were fixed and stained. For the Transwell invasion assay, 80 μL of Matrigel (#356234; Corning, New York, USA) mixed with FBS-free medium was added to the bottom of the upper chamber and allowed to solidify (the ratio of Matrigel:serum-free medium was 1:8), and the other steps were similar to those of the migration assay. Five randomly selected areas were assessed via ImageJ software to determine the number of invasive or migrating cells.

### Wound healing assay

ESCC cells were cultivated in 6-well plates to approximately 90% confluence, after which artificial wounds were created across the bottom of each well using sterilized 200 μL pipette tips. At 0 h, the scratches were imaged via a phase-contrast microscope. Next, the cells were cultured in FBS-free medium for 24 h, and pictures were taken at 24 h via a phase-contrast microscope. ImageJ software was used to quantify cell migration.

### Nuclear‒cytoplasmic extraction

The NE-PER™ Kit (#78833, Thermo Scientific, Massachusetts, USA) was used to isolate the nuclear and cytoplasmic fractions according to the instructions of manufacturer. Proteins localized in the cytoplasmic and nuclear compartments were subsequently assessed via Western blotting.

### Metastasis assay

Lung and liver metastasis models in BALB/c nude mice was utilized to evaluate the in vivo metastatic capacity of ESCC cells (Shanghai SLAC Laboratory Animal Company in China provided the male BALB/c nude mice). Nude mice at 6 weeks of age were randomly classified into two groups as follows (per group: n = 5). Approximately 3 × 10^6^ EC-9706 cells, either transduced with GPR174-overexpressing lentivirus or control lentivirus, were injected into the tail veins of BALB/c nude mice. 4 weeks later (liver metastasis was established for 3 weeks), all the mice were humanely sacrificed. To assess the metastatic potential of ESCC cells, tissues from nude mice were imaged live, stained with hematoxylin and eosin (H&E) and IHC, and weighed. The Animal Welfare Committee of Xinhua Hospital affiliated with Shanghai Jiao Tong University School of Medicine granted permission for conducting ethical animal experiments. All such experiments were meticulously carried out following the directives of the hospital's Animal Welfare Body.

### Statistical analysis

Statistical analysis was performed using SPSS 26.0 and GraphPad Prism 8.0. Unless otherwise stated, paired and unpaired parametric variables were used for Student's t test or one-way ANOVA, and the Mann‒Whitney test was used for nonparametric variables. The data are reported as the means ± SDs. Three repetitions of each experiment were conducted. Statistical significance was determined for differences with p values less than 0.05, Statistical significance in statistical analyses is indicated by ‘*’: * P < 0.05,** P < 0.01,*** P < 0.001,**** P < 0.0001, ns. represents no significance.

## Results

### LysoPS promotes GPR174 expression and is correlated with ESCC invasion and metastasis

**Fig. 1:**
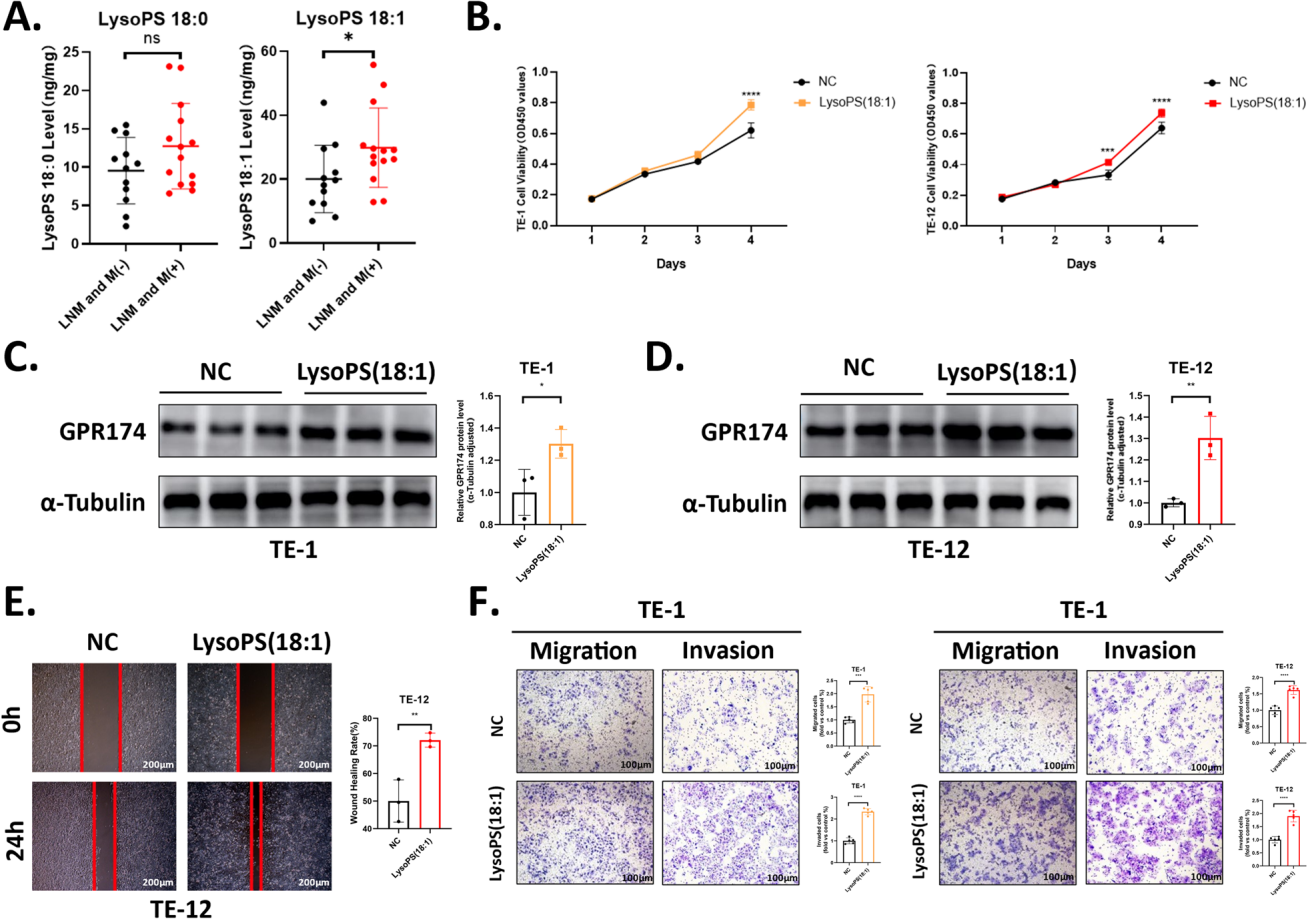
LysoPS promotes GPR174 expression and is correlated with ESCC invasion and metastasis. **A** LC–MS analysis revealed the concentrations of LysoPS (18:0) and LysoPS (18:1) in ESCC tissues. (n = 26) **B** Proliferation of TE-12 and TE-1 cells treated with 10 μM LysoPS (18:1) for 24, 48, 72, and 96 h. **C**, **D** Expression and statistical analysis of GPR174 in TE-1 and TE-12 cells after 1 day of treatment with 10 μM LysoPS (18:1). **E** Cell migration of TE-12 cells assessed by scratch assay following 12 h of treatment with 10 μM LysoPS (18:1), along with its statistical analysis. Scale bars, 200 μm. **F** An assessment of cell invasion and migration in TE-1 and TE-12 cells after 48 h of treatment with 10 μM LysoPS (18:1) using the Transwell assay, along with corresponding statistical analyses. Scale bars, 100 μm

LysoPS (18:1) and LysoPS (18:0) are the types of LysoPS that have been most extensively studied in the context of tumor diseases [11, 24]. To explore the relationship between LysoPS and ESCC, we initially assessed LysoPS levels in tumor tissues from 26 ESCC patients who had not received radiotherapy/chemotherapy prior to surgery or endoscopic resection via LC‒MS technology [25]. Compared with patients without metastasis (including lymph node and distant metastasis), those with metastasis presented elevated levels of LysoPS (18:1), whereas both groups had similar levels of LysoPS (18:0) (Fig. 1A). These results indicate that ESCC metastasis is associated with LysoPS (18:1) levels but not LysoPS (18:0) levels. To further confirm the function of LysoPS(18:1) in facilitating ESCC metastasis, we subsequently stimulated ESCC cells with 10 μM exogenous LysoPS (18:1) in the medium and assessed the proliferation of ESCC cells. As anticipated, LysoPS(18:1) increased the proliferation of ESCC cells (Fig. 1B) [11]. LysoPS can induce the chemotactic migration of glioma cells [26]. We also observed an elevation in the number of ESCC cells undergoing invasion and migration in the LysoPS(18:1)-treated group (Fig. 1E, F). Furthermore, the expression of GPR174 was increased in the LysoPS(18:1)-treated group (Fig. 1C, D). Collectively, these data suggest that LysoPS, which acts upstream of GPR174, can increase GPR174 expression and promotes tumour metastasis in ESCC. Thus, LysoPS/GPR174 may be a key contributor to ESCC metastasis.

### High levels of GPR174 in cancer tissues are associated with a poor prognosis

**Fig. 2:**
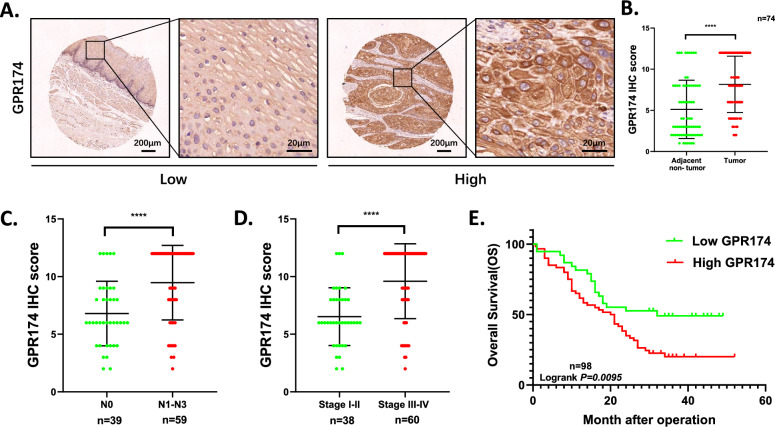
High levels of GPR174 in cancer tissues are associated with a poor prognosis. **A** An illustrative IHC image depicting GPR174 expression in ESCC tissue, with scale bars indicating 200 μm and 20 μm. **B** A statistical evaluation of GPR174 expression in cancerous versus adjacent non-cancerous tissues, derived from IHC data of 74 ESCC patients. **C**, **D** A statistical assessment of GPR174 expression in cancerous tissues from a cohort of 98 ESCC patients, utilizing IHC results of TMA. **E** Kaplan–Meier survival analysis of 98 patients with ESCC presenting high or low GPR174 expression

Building upon our initial demonstration of LysoPS-mediated ESCC invasion and metastasis and its capacity to upregulate GPR174 expression in ESCC cells, we conducted investigations to interrogate the functional contribution of GPR174 in ESCC progression. In a TMA consisting of 98 ESCC tumor samples and 74 matched pairs of ESCC tissues and adjacent normal epithelial tissues, IHC staining was utilized to define the level of the GPR174 protein. Our analysis revealed a distinct upregulation of GPR174 expression in ESCC tissues relative to noncancer tissues (Fig. 2A, B). In the analysis of ESCC tissues from different patients, GPR174 expression was elevated in ESCC tissue with lymph node invasion (N1-3) and classified as stage III/IV compared with ESCC tissues without lymph node invasion (N0) and classified as stage I/II according to the 7th edition American Joint Committee on Cancer (AJCC) staging system (Fig. 2C, D). Using Kaplan‒Meier survival analysis, we evaluated the link between the levels of GPR174 and overall survival (OS) in 98 ESCC patients. A lower level of GPR174 expression was correlated with longer OS compared to patients with a higher level (Fig. 2E).

On the basis of the immunohistochemistry score results for ESCC tissues, the median staining score for GPR174 was 8. Consequently, we designated a score of 8 as the cutoff value for GPR174 expression levels, defining samples with a total score of less than 8 as the low-expression group for GPR174 and samples with a total score of 8 or higher as the high-expression group. Additionally, we assessed the associations between the level of GPR174 and the clinicopathological characteristics of patients with ESCC. GPR174 expression is positively linked to lymph node invasion and tumour–node–metastasis (TNM) stage in ESCC. However, the associations between GPR174 expression level and the age, sex, tumor size, pathological differentiation grade, and T stage of ESCC patients were not statistically significant (Table 1). According to these findings, GPR174 may contribute to ESCC metastasis and lead to poor prognosis.

**Table 1:** Correlation between GPR174 protein expression and clinicopathological features of 98 patients with esophageal squamous cell carcinoma

Clinicopathological variables	n	GPR174 expression		*P* value^a^
		Low	High	
Age				0.102
< 60	30	8	22	
≥ 60	68	30	38	
Gender				0.638
Female	15	5	10	
Male	83	33	50	
Tumor Size				0.290
< 5 cm	58	25	33	
≥ 5 cm	40	13	27	
Differentiation				0.478
Well and moderate	71	26	45	
Poorly and not	27	12	15	
TNM stages^b^				< 0.0001*
I–II	38	24	14	
III–IV	60	14	46	
Tumor invasion				0.071
T1–T2	13	8	5	
T3–T4	85	30	55	
Lymphatic invasion				< 0.001*
N0	39	23	16	
N1–N3	59	15	44	

^a^All P values were determined by chi-square test; ^b^TNM staging was AJCC 7th edition staging

### GPR174 drives the epithelial–mesenchymal transition (EMT) and metastasis of ESCC cells

**Fig. 3:**
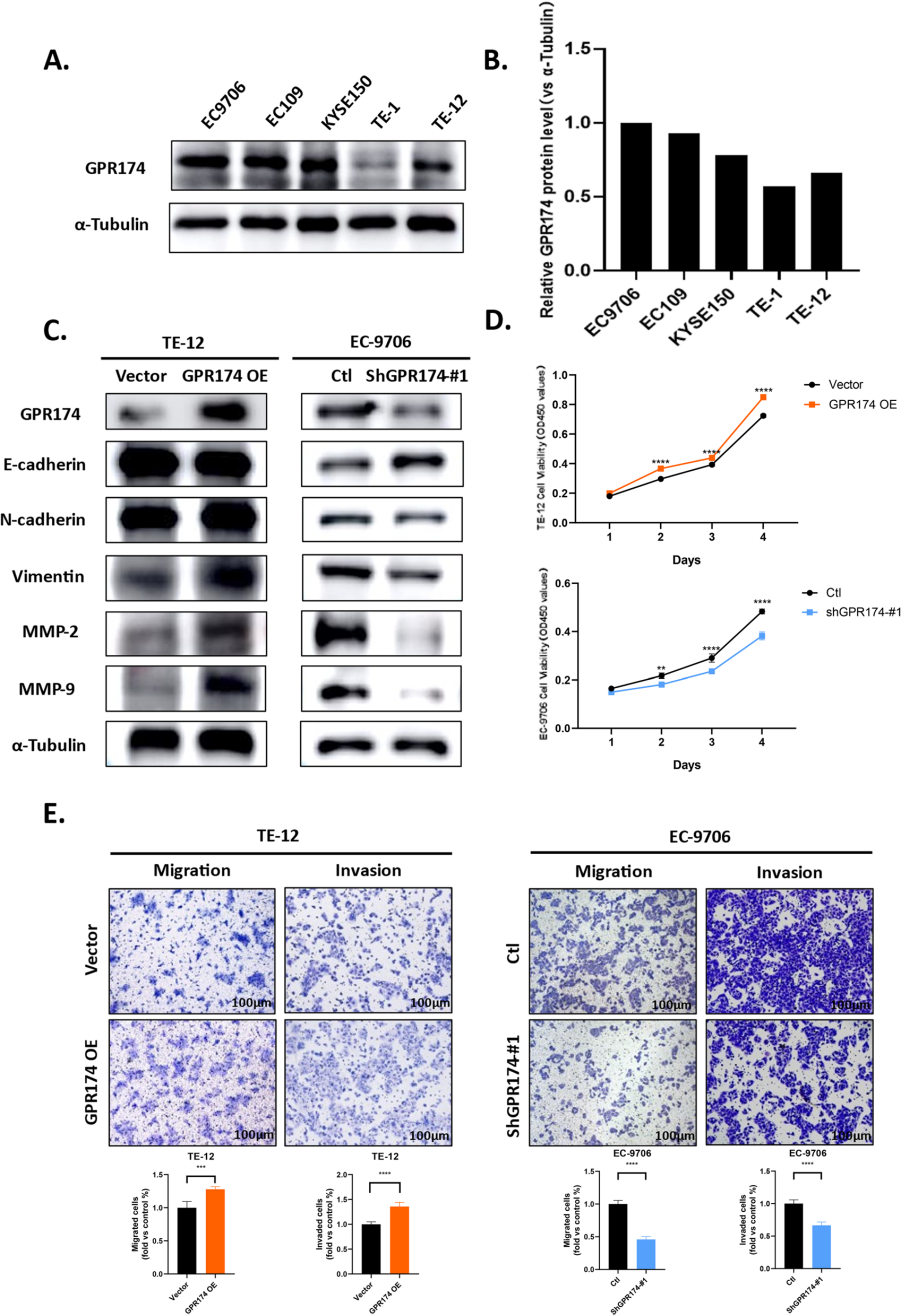
GPR174 drives the EMT and metastasis of ESCC. **A**, **B** Western blot analysis reveals the levels of GPR174 in various ESCC cell lines along with corresponding statistical analysis. **C** Overexpression of GPR174 in TE-12 cells and knockdown of GPR174 in EC-9706 cells result in altered levels of GPR174, E-cadherin, N-cadherin, Vimentin, MMP-2, and MMP-9. **D** Cell proliferation in GPR174-overexpressing and GPR174-knockdown ESCC cells is examined using the CCK-8 assay. **E** Representational images and statistical analysis of the Transwell migration/invasion assays performed on GPR174-overexpressing TE-12 cells and GPR174-knockdown EC-9706 cells. Scale bars, 100 μm

To further dissect the molecular mechanisms by which GPR174 promotes the invasion and metastasis of ESCC, we profiled the baseline expression of GPR174 in various ESCC cells to discover the associations of GPR174 with the EMT and metastasis of ESCC cells. Quantitative analysis revealed heterogeneous GPR174 expression in ESCC cell lines. The protein basal expression levels of GPR174 were greater in the EC-9706, EC-109, and KYSE-150 cell lines than in the TE-12 and TE-1 cell lines (Fig. 3A, B). TE-12 cells exhibiting constitutively low GPR174 levels were engineered to overexpress this receptor via lentiviral transduction. In the EC9706 cell line, which has high GPR174 expression, GPR174 was successfully knocked down. Moreover, for the EC-9706 cells, we observed greater knockdown efficiency in the shGPR174-#1 group than in the shGPR174-#2 and shGPR174-#3 groups (Fig. S2). Next, we sought to investigate the functional role of GPR174 in ESCC progression through cells transduced with GPR174 lentivirus. GPR174 overexpression promoted TE12 cell proliferation, whereas GPR174 knockdown reduced the proliferative capacity of EC-9706 cells (Fig. 3D).

EMT, a complex biological process by which epithelial cells transform into mesenchymal cells, is considered a key feature of cancer progression [27]. EMT is indicated by altered the levels of proteins such as E-cadherin, N-cadherin and vimentin. It is crucial for cancer invasion and metastasis that the extracellular matrix (ECM) plays a role [28]. Collagen constitutes 90% of the ECM [29]. The gelatinolytic enzymes matrix metalloproteinase-2 (MMP-2) and MMP-9 are collectively termed gelatinases and are consistently linked to the progression of solid tumors through their roles in invasion, metastasis, and angiogenesis [30]. Collagen type IV and denatured collagen are among the ECM components that can be degraded by MMP-2 and MMP-9. The degradation of collagen may facilitate tumor metastasis and invasion, thereby influencing the progression of malignant tumors [31, 32]. The occurrence of invasion and metastasis in ESCC is inseparable from the EMT process and the degradation of the ECM by matrix metalloproteinases. Western blotting was utilized to measure the expression of EMT-related proteins and MMP-9 and MMP-2. We found that a significant increase in N-cadherin, vimentin, MMP-2, and MMP-9 expression was observed when GPR174 was overexpressed, while reduced levels of E-cadherin were detected. Conversely, in the GPR174 knockdown group, the opposite trend was observed (Fig. 3C). Consequently, experimental validation of our hypothesis revealed that GPR174 orchestrates the EMT program and drives ECM remodeling. According to the results of the Transwell assays, compared with the control, GPR174 overexpression significantly promoted TE-12 cell migration and invasion. Conversely, knockdown of GPR174 suppressed the invasion and migration of EC-9706 cells (Fig. 3E).

### GPR174 contributes to the metastasis of ESCC through the cAMP-PKA-CREB axis

**Fig. 4:**
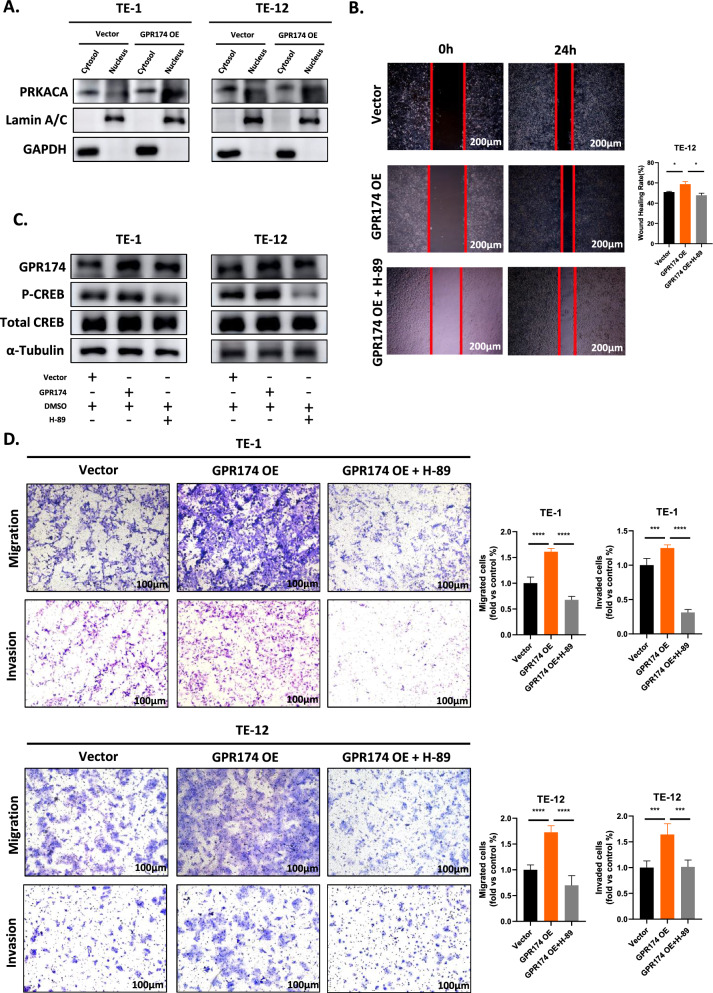
GPR174 contributes to the metastasis of ESCC through the cAMP-PKA-CREB axis. **A** Nuclear-cytoplasmic extraction assays and Western blot analyses were applied to quantify the expression of PRKACA in both the nuclear and cytoplasmic of the cells. **B** The impact of a 24h treatment with 10 μM H-89 (dissolved in DMSO) on the migratory capacity of TE-12 cells overexpressing GPR174 was illustrated, along with the corresponding statistical analysis. Scale bars, 200 μm. **C** Typical images of Western blotting are shown, demonstrating the expression levels of GPR174, phosphorylated CREB (P-CREB), and total CREB (Total-CREB) in ESCC cells overexpressing GPR174 after a 48h exposure to 10 μM H-89 (dissolved in DMSO). **D** Statistical analyses and typical images of the Transwell migration and invasion assays for ESCC cells, following a 48h treatment with H-89 (dissolved in DMSO), are presented. Scale bars, 100 μm

Currently, the role and underlying mechanisms of GPR174 in ESCC are unknown. Several previous studies have indicated that GPR174 can activate cAMP and its downstream signaling molecule PKA by coupling with Gαs [7, 14, 33]. In the absence of cAMP activation, the PKA tetramer remains inactive. But upon activation, PKA releases its active subunit, PRKACA, which subsequently agonizes and phosphorylates downstream signaling molecules [34]. Importantly, CREB, a downstream signaling molecule of PKA, has been shown to facilitate the invasive and metastatic processes of ESCC upon its activation [22]. We hypothesize that GPR174 may regulate the phosphorylation levels of CREB in ESCC cells through the modulation of cAMP/PKA activation, thereby facilitating ESCC metastasis. To investigate this hypothesis, we employed a nucleocytoplasmic fractionation assay to evaluate the activation of PRKACA and its localization within TE-1 and TE-12 cell lines that overexpress GPR174. Our results revealed that GPR174 overexpression did not significantly alter cytoplasmic PRKACA levels, but there was a marked increase in nuclear PRKACA. This finding suggests that GPR174 activates the cAMP/PKA pathway and that the active subunits of PKA, once activated, predominantly translocate to the nucleus to exert their effects (Fig. 4A). To investigate the function of CREB in ESCC cell invasion and metastasis, we exposed TE-1 and TE-12 cells overexpressing GPR174 to the PKA-selective inhibitor H-89 at a concentration of 10 μM to inhibit PKA activation. Furthermore, we investigated whether ESCC cells had increased invasiveness and metastatic potential and assessed CREB phosphorylation status [35]. The results revealed that PKA inhibitor suppressed CREB phosphorylation and clearly reduced the invasive and metastatic potential of GPR174-overexpressing ESCC cells (Fig. 4B–D). These findings demonstrate that CREB, a downstream signaling molecule of PKA, is manipulated by the GPR174/cAMP/PKA axis and is positively correlated with ESCC metastasis. These results illustrate that GPR174 may promote ESCC metastasis via the cAMP-PKA-CREB axis.

### In vivo study of the function by which GPR174 promotes ESCC metastasis

**Fig. 5:**
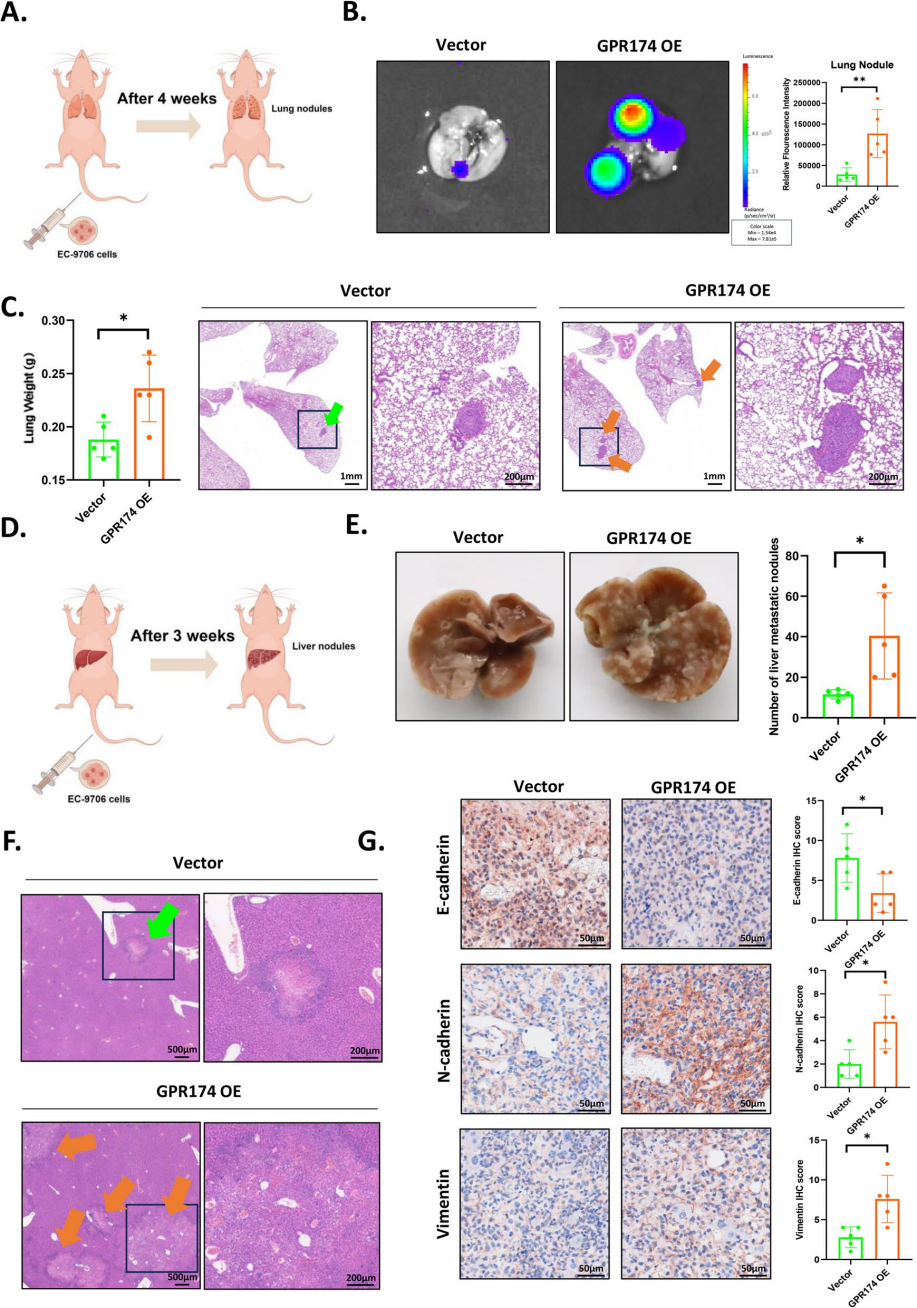
In vivo study of the function by which GPR174 promotes ESCC metastasis. **A** An illustration of a model for establishing lung metastases in nude mice. **B** Typical images of in vivo imaging of lung tissues from nude mouse, along with statistical analysis (per group: n = 5).** C** Statistical analysis of lung weight (per group: n = 5) and typical H&E staining images of lung metastasis samples. Scale bars, 1 mm and 200 μm. **D** An illustration of a model for establishing liver metastases in nude mice. **E** Representative images of metastatic tumors on the surface of nude mouse liver, along with statistical analysis (per group: n = 5). **F** Typical H&E staining images of liver metastasis samples. Scale bars, 500 μm and 200 μm. **G** Typical IHC staining images of EMT biomarker (E-cadherin, N-cadherin and vimentin) in liver metastasis samples. Scale bars, 50 μm

Through clinical sample analysis and molecular biology experiments, we confirmed the involvement of GPR174 in ESCC invasion and metastasis. The most common organs affected by distant metastases from ESCC are the lungs and liver, followed by the bones and brain [36, 37]. Accordingly, to further validate this conclusion, we established experimental models of pulmonary and hepatic metastasis (Fig. 5A, D). Living imaging experiments revealed that, the GPR174-overexpressing nude mice showed increased lung metastasis in comparison with the control group (Fig. 5B). Subsequently, we observed an increase in metastasis in the GPR174 overexpression group in terms of the number of metastatic nodules (Fig. 5C, E–F). Given the pivotal role of EMT remodeling in tumor metastasis, we next interrogated the impact of GPR174 on EMT biomarker expression (E-cadherin, N-cadherin and vimentin) within hepatic metastasis models. Immunohistochemical profiling revealed significant downregulation of E-cadherin concomitant with upregulated N-cadherin and Vimentin expression in GPR174-overexpressing metastatic lesions, in full concordance with our in vitro findings (Fig. 5G). Collectively, these in vivo data functionally validate the pro-metastatic capacity of GPR174 through EMT reprogramming in ESCC progression.

## Discussion

ESCC has a 5-year survival rate of less than 20%, mainly because of its high recurrence and metastasis risk and acquired drug resistance [38]. Esophageal carcinoma is notably prone to lymphatic dissemination. Although squamous cell carcinoma is the predominant histological subtype of esophageal malignancies, the intricate pathways of its invasive and metastatic progression have yet to be fully elucidated [39–41]. The identification of specific targets has increased the precision of cancer therapy. LysoPS and its receptor, GPR174, serve as crucial modulators in immune and inflammatory diseases [42]. While LysoPS has been involved in the invasion and metastasis of various cancers, LysoPS/GPR174 remains a mystery in terms of its function and mechanisms of action in ESCC progression. In previous studies, GPR174 was found mainly in lymphoid organs such as the spleen, thymus, and lymph nodes [5]. Our study provides the first evidence that LysoPS/GPR174 is critical for facilitating the invasion and metastasis of ESCC. We discovered that the metastasis of ESCC is associated with the expression of LysoPS (18:1) in ESCC tissues, whereas no such association was observed for LysoPS (18:0). Moreover, LysoPS(18:1) has been shown to induce the expression of GPR174 in ESCC.

**Fig. 6:**
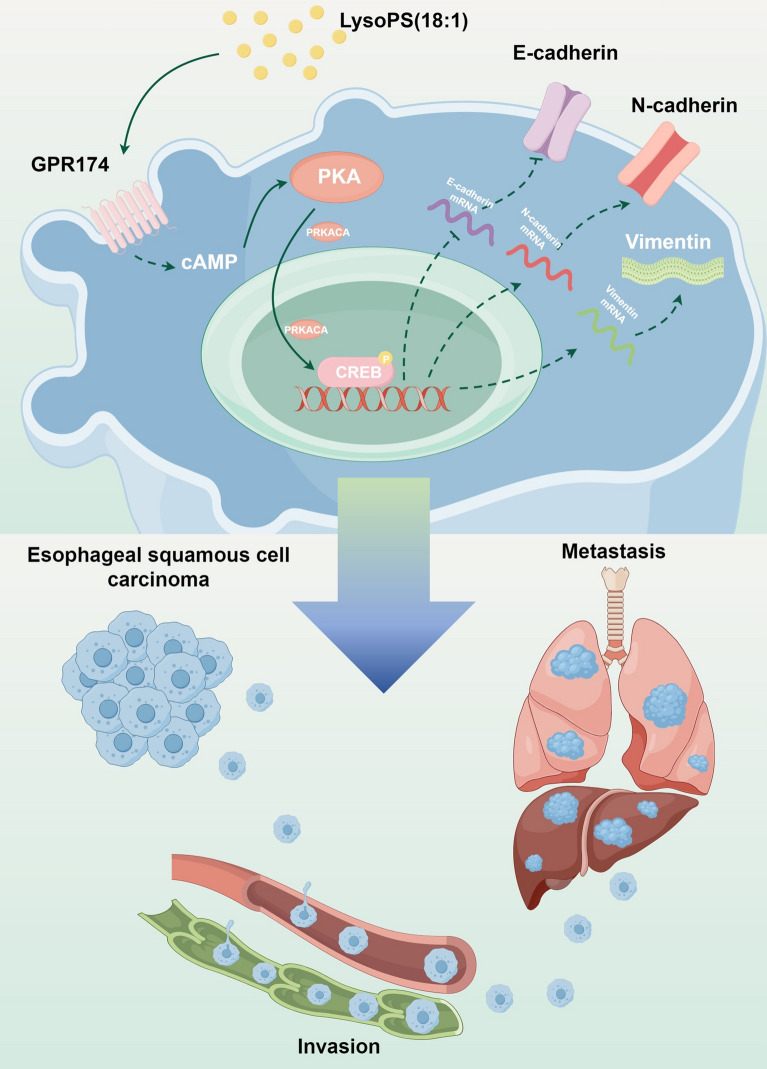
LysoPS/GPR174 stimulates esophageal squamous cell carcinoma invasion and metastasis via the cAMP-PKA-CREB pathway

Our study subsequently revealed elevated levels of GPR174 expression in ESCC patients with lymph node invasion and advanced AJCC stage. Furthermore, patients with GPR174 overexpression had reduced OS. ESCC patients may benefit from the use of GPR174 as a metastatic marker and prognostic indicator. These findings suggest that LysoPS (18:1) may facilitate the invasion and metastasis of ESCC by promoting GPR174 expression on the surface of ESCC cells. To validate the involvement of GPR174 in the invasion and metastasis of ESCC, we conducted molecular biology experiments and animal studies and revealed that GPR174 promotes the EMT process and the occurrence of invasion and metastasis in ESCC. The GPCR family responds to a variety of extracellular signals by modulating G proteins, and GPR174, a member of this family, functions in a similar way [43]. We further investigated the signaling mechanisms by which GPR174 promotes ESCC metastasis. Our results revealed that GPR174 can increase the metastatic potential of ESCC through the cAMP-PKA-CREB signaling pathway (Fig. 6).

In conclusion, our study illustrates for the first time that GPR174 contributes to ESCC invasion and metastasis in response to LysoPS stimulation. This investigation has yielded valuable insights that may improve clinical diagnostic methods and therapeutic interventions for ESCC.

## Supplementary Information

Supplementary material 1: **Fig. S1.** Scoring criteria for immunohistochemical findings in TMA of ESCC. **A** Example of ESCC tumour tissue and adjacent normal epithelial tissue. **B** The percentage of positively stained cells was divided into five categories: <5% was 0, 6-25% was 1, 26-50% was 2, 51-75% was 3, and >75% was 4. **C** The intensity of immunohistochemical staining was divided into five categories according to conventional criteria: negative was 0; weak was 1; moderate was 2; or strong was 3. **Fig. S2.** Overexpression and knockdown of ESCC cell lines infected with GPR174 lentivirus. **Table S1.** Core sequences of lentivirus about shGPR174. **Table S2.** Primers used for GPR174 genes amplification.

## Data Availability

Upon reasonable request, the corresponding author will provide the source data.

## Abbreviations

ESCC: Esophageal squamous cell carcinoma
GPCR: G protein-coupled receptor
LysoPS: Lysophosphatidylserine
Gαs: G protein alpha subunit
cAMP: Cyclic adenosine monophosphate
PKA: Protein Kinase A
CREB: Cyclic AMP response element-binding protein
FBS: Fetal bovine serum
TMA: Tissue microarray
H-89: *N*-[2-(P-bromocinnamylamino)ethyl]-5-isoquinolinesulfonamide·2HCl hydrate
DMSO: Dimethyl sulfoxide
LC‒MS: Liquid chromatography–mass spectrometry
AJCC: American Joint Committee on Cancer
OS: Overall survival
TNM: Tumour–node–metastasis
EMT: Epithelial–mesenchymal transition
ECM: Extracellular matrix
MMP-2: Matrix metalloproteinase-2
