# Supplementary material for: The LysoPS/GPR174 axis drives metastatic progression in esophageal squamous cell carcinoma through cAMP-PKA-CREB signaling activation
Source: J Transl Med. 2025 Apr 14;23:438. doi: 10.1186/s12967-025-06419-0 (PMC11995483; doi:10.1186/s12967-025-06419-0)
Supplement: Supplementary file 1 — Supplementary material 1: Fig. S1. Scoring criteria for immunohistochemical findings in TMA of ESCC. A Example of ESCC tumour tissue and adjacent normal epithelial tissue. B The percentage of positively stained cells was divided into five categories: <5% was 0, 6-25% was 1, 26-50% was 2, 51-75% was 3, and >75% was 4. C The intensity of immunohistochemical staining was divided into five categories according to conventional criteria: negative was 0; weak was 1; moderate was 2; or strong was 3. Fig. S2. Overexpression and knockdown of ESCC cell lines infected with GPR174 lentivirus. Table S1. Core sequences of lentivirus about shGPR174. Table S2. Primers used for GPR174 genes amplification. [file 12967_2025_6419_MOESM1_ESM.docx]

**Supplementary Materials**

**Figure S1**


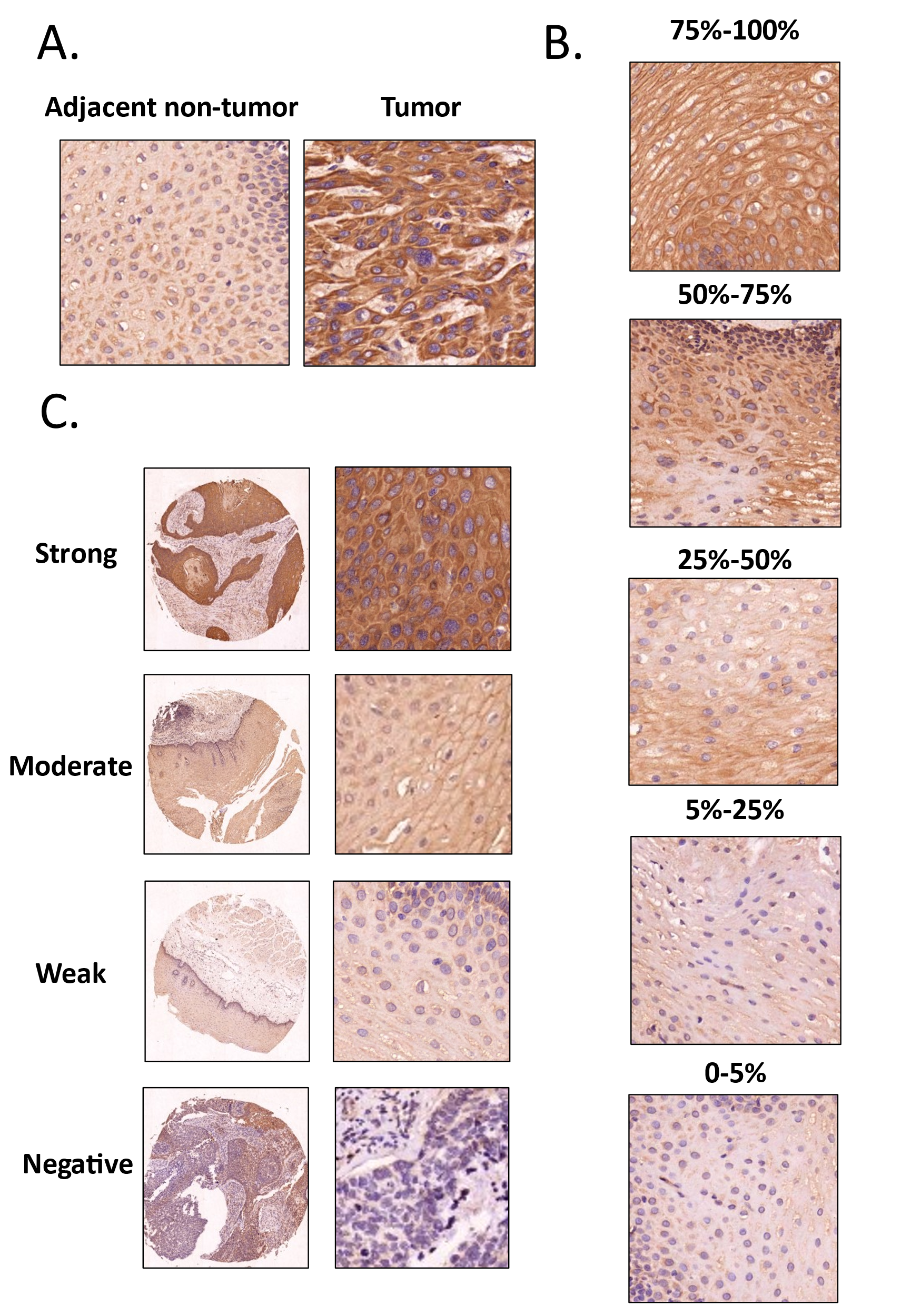


**Fig. S1** Scoring criteria for immunohistochemical findings in TMA of ESCC

**A.** Example of ESCC tumour tissue and adjacent normal paracancerous epithelial tissue.

**B.** The percentage of positively stained cells was divided into five categories: <5% was 0, 6-25% was 1, 26-50% was 2, 51-75% was 3, and >75% was 4. **C.**The intensity of immunohistochemical staining was divided into five categories according to conventional criteria: negative was 0; weak was 1; moderate was 2; or strong was 3.


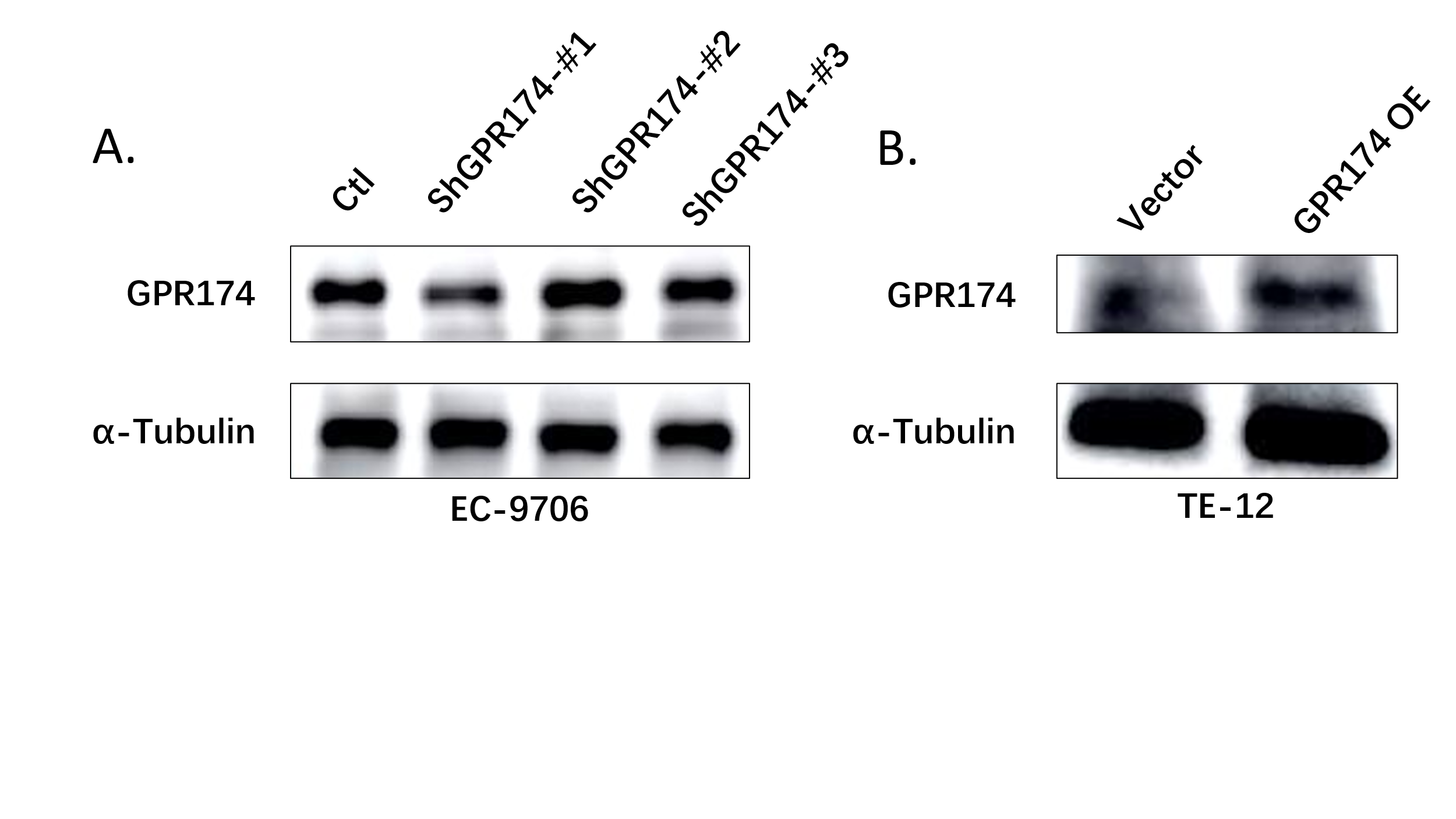
**Figure S2**

**Fig. S2** Overexpression and knockdown of ESCC cell lines infected with GPR174 lentivirus

**Table S1** Core sequences of lentivirus about shGPR174

| Target gene | Sequences |
| --- | --- |
| Ctl | 5’-TTCTCCGAACGTGTCACGT-3’ |
| shGPR174-#1 | 5’-CCACCATGACACCTGAATTAT-3’ |
| shGPR174-#2 | 5’-CGCTTCTGATTGTCCTATATT-3’ |
| shGPR174-#3 | 5’-GCTGACTTACTACAAGTTCTT-3’ |

**Table S2** Primers used for GPR174 genes amplification

| Target gene | Sequence(5’-3’) |
| --- | --- |
| GPR174 | **FW:**CCAACTTTGTGCCAACCGGTCGCCACCATGCCTGCTAATTACACGTGTACCAGG  **REV:**CACACATTCCACAGGAATTTTAGCATAATTCAGGTGTCATGG |
